# Prospective evaluation of direct approach with a tablet device as a strategy to enhance survey study participant response rate

**DOI:** 10.1186/1756-0500-5-605

**Published:** 2012-10-31

**Authors:** Melissa J Parker, Asmaa Manan, Sara Urbanski

**Affiliations:** 1 Division of Critical Care, Department of Pediatrics, McMaster Children’s Hospital and McMaster University, 1200 Main St W. Room 3A, Hamilton, Toronto, Ontario, L8N 3Z5, Canada; 2 Division of Emergency Medicine, Department of Pediatrics, The Hospital for Sick Children and The University of Toronto, 1200 Main St W. Room 3A, Hamilton, Toronto, Ontario, L8N 3Z5, Canada

**Keywords:** Research design, Data collection, Computers, Handheld, Bias (epidemiology)

## Abstract

**Background:**

Investigators conduct survey studies for a variety of reasons. Poor participant response rates are common, however, and may limit the generalizability and utility of results. The objective of this study was to determine whether direct approach with a tablet device enhances survey study participant response rate and to assess participants’ experiences with this mode of survey administration.

**Findings:**

An interventional study nested within a single center survey study was conducted at McMaster Children’s Hospital. The primary outcome was the ability to achieve of a survey study response rate of 70% or greater. Eligible participants received 3 email invitations (Week 0, 2, 4) to complete a web-based (Survey Monkey) survey. The study protocol included plans for a two-week follow-up phase (Phase 2) where non-responders were approached by a research assistant and invited to complete an iPad-based version of the survey. The Phase 1 response rate was 48.7% (56/115). Phase 2 effectively recruited reluctant responders, increasing the overall response rate to 72.2% (83/115). On a 7-point Likert scale, reluctant responders highly rated their enjoyment (mean 6.0, sd 0.83 [95% CI: 5.7-6.3]) and ease of use (mean 6.7, sd 0.47 [95% CI: 6.5-6.9]) completing the survey using the iPad. Reasons endorsed for Phase 2 participation included: direct approach (81%), immediate survey access (62%), and the novelty of completing a tablet-based survey (54%). Most reluctant responders (89%) indicated that a tablet-based survey is their preferred method of survey completion.

**Conclusions:**

Use of a tablet-based version of the survey was effective in recruiting reluctant responders and this group reported positive experiences with this mode of survey administration.

## Findings

### Background

Survey-based research is commonly undertaken to gain knowledge and understanding of the attitudes, preferences and self-reported practices of individuals within a population [1]. From a clinical research perspective, survey data can provide valuable information to investigators prior to undertaking further study in a particular area. Results obtained from a well-conducted survey study can inform investigators on a number of important feasibility considerations, including level of interest in a topic, current practice patterns, intervention(s) acceptable to practicing clinicians, and willingness to enroll participants into a study [2].

A major hurdle in conducting survey-based research is obtaining an adequate response rate from among those invited to participate. A good response rate, considered 70% or higher, is important to minimize the risk of selection bias [3,4]. Guarding against selection bias is important to increase confidence that the study results are generalizable to the entire population surveyed [5]. Previous work has demonstrated that survey non-respondents may differ from respondents in important ways [6]. Investigators should therefore use all reasonable means to encourage as many eligible subjects as possible to participate in any survey study conducted.

Researchers frequently opt for a survey study design involving postal and/or electronic distribution of self-administered surveys for reasons of cost and convenience [7,8]. Factors known to impact on survey study response rates include survey length, appearance, pre-notification, inclusion of monetary or non-monetary incentives, academic sponsorship, and follow-up [9]. Beyond strategies previously demonstrated to improve survey study response rate, investigators may consider using other creative means to engage and recruit eligible participants [10,11]. In this paper we describe use of a tablet device to enhance participant response rate in a single center survey study involving a self-administered questionnaire.

### Methods

A single center survey study was conducted at McMaster University Medical Centre (Hamilton, Canada) between January and May 2012. The purpose of the survey study was to assess health care providers’ attitudes and beliefs regarding pediatric fluid resuscitation practices. The sampling frame included all nursing and physician staff of the Pediatric Emergency Department (PED) and Pediatric Critical Care Unit (PCCU) of McMaster Children’s hospital (n = 115). We report here the results of a nested study, which prospectively evaluated the effectiveness of direct approach with a tablet-based version of the survey to improve the response rate and participants’ experiences with this approach to survey administration.

For the primary outcome of this nested study, we sought to determine whether use of a recruitment strategy involving a tablet-based version of the survey was effective in improving the response rate to the desirable 70%-plus level within a 2-week time frame. Secondary outcomes related to the assessment of respondents’ enjoyment, ease of use, and experience of any technical problems while using the tablet to complete the survey. We also planned to examine differences in participant characteristics according to study phase and to compare the characteristics of responders and non-responders.

The Faculty of Health Sciences/Hamilton Health Sciences Research Ethics Board approved this study and all procedures were conducted in accordance with the Tri-council Policy Statement [12]. The full study protocol called for the survey to be administered in two distinct phases. In Phase 1, eligible participants were invited to complete the survey at 0, 2, and 4 weeks via an email invitation that included a hyperlink to a web-based (Survey Monkey) version of the survey. An electronic version of the study information and consent sheet was provided as an attachment along with the study invitation email. The email invitation advised eligible participants of an incentive (chance to win a coffee card of $25-value). Participants were instructed to complete the survey only once. The protocol included provisions to proceed with a second phase of survey distribution should a response rate of 70% or more not be achieved by 1 month following the final email inviting survey participation.

In Phase 2 a pre-notification email was sent to all individuals in the sampling frame advising that a research assistant would be visiting the PED and PCCU over a 2-week period to offer a final opportunity to complete the survey. Potentially eligible subjects were approached in person by a research assistant and offered a paper copy of the information and consent sheet inviting study participation. Those approached were advised of the participation incentive, which was the same as that offered during Phase 1. Individuals were considered potentially eligible subjects if they were present and/or working in the relevant clinical environment and appeared available to be approached. Interested persons were screened for eligibility and consent was obtained from qualifying individuals who had not previously completed the survey. Eligible subjects were informed that they were being invited to complete an iPad-based version of the survey.

The research assistant presented consenting participants with an Apple iPad2 tablet device, trademark of Apple Inc. (32 GB with Wi-Fi, Apple model A1395), to facilitate completion of the tablet-based version of the survey. The iPad-based version of the survey was created using a web-based program, iSURVEY (iSurveySoft, Wellington, NZ), and administered using an iPad application by the same name. This survey program differs from the Survey-Monkey program in that iSURVEY facilitates transfer of the survey onto the iPad with appropriate formatting. This application also permits access to and completion of the survey when the iPad is not connected to the Internet, allowing the survey to be iPad-based rather than web-based. Completed surveys are stored on the iPad until an Internet connection is reestablished, at which time, completed surveys are uploaded to the company website. Completed surveys located (temporarily) on the iPad cannot be accessed once completed and are fully secure. This program allows for many surveys to be completed using an iPad while not connected to the Internet and then subsequently uploaded at a later time that is convenient. This significantly increases the flexibility of survey administration, as the iPad is small and portable. Further details regarding the nature of this application can be found on the company website, https://www.isurveysoft.com.

The Phase 2 version of the survey was modified in that 5 questions eliciting participants’ experience completing the iPad-based version of the survey were included following the original slate of 15 questions. On a 7-point Likert scale, participants were asked to rate their level of enjoyment (1 = no enjoyment; 4 = neutral; 7 = it was an amazing experience) and ease of use (1 = it was extremely difficult; 4 = neutral; 7 it was extremely easy) completing the iPad-based version of the survey. A free text question also asked whether respondents experienced any technical problems while attempting to complete the survey. Given that our Phase 2 respondents were reluctant responders, we included questions asking why they elected to participate at this time but not when previous invitations had been sent. We also asked them to report their preferred method of survey completion.

The primary outcome analysis consisted of determination of achievement of a 70% response rate based on the calculated proportion of surveys completed. Descriptive data regarding incomplete surveys and item response rate are reported. Binary logistic regression was used to obtain the odds ratio point estimate for submission of incomplete surveys in Phase 1 as well as the odds ratio point estimate for missing item responses. Participant characteristics and survey data are summarized using mean (standard deviation) for continuous variables and count (percent) for categorical variables, with 95% confidence intervals (CI) included where appropriate. Binary logistic regression was used to generate univariate odds ratio point estimates and 95% confidence intervals for participant characteristics, with Phase 1 survey completion as the dependent variable. We planned *a priori* to include significant variables into a multivariable model to determine independent effects, if appropriate. In all cases, significance was determined at the p < 0.05 level. Collected survey data were analyzed using IBM SPSS statistics, Version 20 (IBM, Armonk, New York, USA). Data related to non-respondent characteristics were analyzed using an on-line calculator for 2 x 2 contingency tables [13].

### Results

At completion of Phase 1, the survey study response rate was 56/115 (48.7%). An additional 27 eligible participants completed the survey during Phase 2 yielding a final study response rate of 83/115 (72.2%). One eligible participant declined study participation when directly approached. Table 1 illustrates the cumulative participant response rate by study phase.

**Table 1:** Survey response rate by study phase

**Survey Study Phase**	**Number of Participant Responses Received**	**Cumulative Number of Participant Responses Received and Response Rate (%)**
**Phase 1**		
Week 0-2	28	28 (24.3)
Week 2-4	12	40 (34.8)
Week 4-10	16	56 (48.7)
**Phase 2**		
Week 10-11	16	72 (62.6)
Week 11-12	11	83 (72.2)

Phase 1 - Email invitation/Self-administered web-based survey.

Phase 2 - Direct approach/Self-administered tablet-based survey.

The characteristics of Phase 1 and Phase 2 study participants are displayed in Table 2. Only increasing pediatric resuscitation experience was found to be significantly associated with Phase 1 survey completion. Item response rate was high, with 1234/1245 (99.1%) of questions answered. The proportion of answered questions according to study phase was 833/840 (99.2%) for Phase 1 and 401/405 (99.0%) for Phase 2, yielding an odds ratio point estimate of 0.84 [95% CI: 0.22-3.43] for missing items in Phase 1. Eleven percent (6/56) of participants submitted surveys with missing items in Phase 1 compared to 3.7% (1/27) in Phase 2. The odds ratio point estimate for submission of an incomplete survey in Phase 1 was 3.12 [95% CI: 0.34-72.5]).

**Table 2:** Participant characteristics by survey study phase

**Participant Characteristics**	**Phase 1 N(%)**	**Phase 2 N(%)**	**Odds Ratio Point Estimate [95% CI]**
Profession			^a^7.56 [0.93-61.28]
Nurse	43 (77)	25 (96)	
Staff Physician or Fellow	13 (23)	1 (4)	
Primary Area of Practice			^b^0.82 [0.32-2.11]
Pediatric Emergency Medicine	21(38)	11(42)	
Pediatric Critical Care Medicine	35 (62)	15 (58)	
Work Status			^c^0.37 [0.10-1.43]
Full Time	40 (74)	23 (88)	
Part Time or Occasional Staff	14 (26)	3 (12)	
Years of Experience in Pediatric Emergency of Pediatric Critical Care Medicine			^d^1.29 [0.87-1.92]
Less than 2 years	11 (20)	9 (33)	
2-5 years	16 (28)	7 (26)	
5-9 years	9 (16)	4 (15)	
10 years or more	20 (36)	7 (26)	
Pediatric Resuscitation Experience*			^e^1.76 [1.073-2.90]
None or Minimal experience	4 (7)	9 (33.3)	
Some experience	17 (30)	6 (22.2)	
Experienced or Very Experienced	35 (66)	12 (44.4)	

Univariate odds ratio point estimates generated using binary logistic regression.

a – staff physician or fellow profession.

b – pediatric emergency medicine.

c – full time staff.

d – increasing years of experience.

e – increasing resuscitation experience.

The limited data available regarding non-respondents reveals that these individuals were more likely to work as part time or occasional staff (17/34 (50.0%) vs. 18/81 (22.2%), odds ratio point estimate 3.5 [95% CI: 1.49-8.21]. Physicians were no less likely to be non-responders when compared to nurses (7/21 (33.3%) vs. 26/94 (27.7%), odds ratio 1.31, [95% CI: 0.42-3.99]) and the odds of non-response was not significantly influenced by primary area of practice (19/51 (37.3%) for PED vs. 14/64 (21.9%) for PCCU, odds ratio 2.12 [95% CI: 0.87-5.23]).

Participants who completed the tablet-based version of the survey reported a positive experience. The mean (sd) level of enjoyment rating was 6.0 (0.83) [95% CI 5.7-6.3] while the mean (sd) rating for ease of use was 6.7 (0.47) [95% CI 6.5-6.9]. No technical problems were reported. In fact, many participants actually used this free text section to provide additional positive feedback regarding their experience with this mode of survey administration, e.g. “None – all surveys should be like this!” Reasons reluctant responders endorsed for participating in the study during Phase 2 are listed in Table 3 while preferences regarding mode of survey administration are listed in Table 4.

**Table 3:** Reasons endorsed by reluctant responders regarding why they elected to participate during Phase 2

**Factors Influencing Study Participation**	**Participants Selecting Item N (%)**
Being approached/invited in person was important	21 (81)
Ability to immediately access the survey was important	16 (62)
The novelty of completing the survey using the iPad	14 (54)
The fact that internet access was not required	3 (12)
I tend to delete any email that invites survey study participation	7 (27)

Description: Of the 27 reluctant responders who completed the survey during Phase 2, 26 answered the question, “Indicate which factors influenced you to participate in this survey study now compared to when you were previously invited to participate by email”. More than one response could be selected.

**Table 4:** Preferred mode of survey administration of reluctant responders

**Survey administration method**	**Number of respondents selecting N (%)**
Hard Copy (Pen and Paper)	0
Internet/Web-based survey e.g. Survey Monkey	3 (11)
Tablet Device e.g. iPad	24 (89)
Smart Phone Device e.g. iPhone	0
Telephone (Verbal) Interview	0
Face-to-Face (In person, verbal) Interview	0

Description: Of the 27 reluctant responders who completed the survey during Phase 2, 27 answered the question, “Please indicate your preferred method for completing a survey”. Only one response could be selected.

### Discussion

An ongoing challenge for investigators is to find effective ways to engage potential study participants in survey-based research. Recent data suggests that survey response rates are in fact deteriorating [14], and that surveys of physicians in particular are at risk for low response rates [5,15-18]. While monetary incentives appear to be effective in increasing response rates [9,17,18], this can add substantially to study costs depending on the value of the incentive and the number of participants surveyed. In this single center survey study of physician and nursing staff we demonstrate that direct approach with a tablet device is an effective strategy to increase participant response rate into the desirable 70%-plus range and that this mode of survey administration is well received by reluctant responders.

To our knowledge, this is the first report describing use of a tablet device as a tool to enhance participant response rate in a survey study of health care professionals. A search of OVID MEDLINE (R) 1946 to June Week 1, 2012, under the MeSH heading “Computers, Handheld” revealed 1783 results, with 64 unique citations also mapping to the “Data Collection” MeSH heading. Published reports describe a variety of types and uses of Personal Digital Assistants (PDAs) for the purpose of data collection including use in remote fieldwork [19-23], household surveys [22,24], patient data diaries [25-28], observational research [29-32], quality assurance [33,34], and laboratory data collection [35]. Good agreement has been found between paper-based and PDA-based survey data [36-38], with PDA-based methods yielding comparatively greater data accuracy and less missing data [36,39].

In the present study, several findings are of interest in addition to the primary outcome result. First, the resuscitation experience of respondents significantly differed by study phase, suggesting that individuals with greater confidence in the survey subject matter were more likely to participate when initially invited. Secondly, there was a trend toward increased recruitment of physicians in Phase 1 of the study. While non-significant, the odds ratio point estimate was more than seven, with a wide confidence interval owing to our small sample size. It is possible that participating physicians may have been more inclined to complete the survey during Phase 1 due to their level of experience and comfort with the subject matter. Conversely, the research assistant may have had more difficulty accessing physicians or felt less comfortable approaching them to invite study participation during Phase 2. Lastly, direct approach, immediate survey access, and the novelty of this survey strategy were endorsed by a majority of reluctant responders as aiding in their recruitment. Given that most reluctant responders indicated that a tablet-based mode of survey completion is their preference, this strategy should be further evaluated in the context of a larger study of health care providers.

It will be important to clarify in future work that use of this strategy actually improves representativeness and does not exacerbate response bias. Direct approach with a tablet device resulted in the recruitment of units that otherwise would have been missing, and our data indicate that some differences in participant characteristics existed between the two study phases. This, however, does not answer the question of whether the data that would have been missing (or remains missing) is missing at random (MAR) or not, which is the major determinant of whether survey results are representative of the total population surveyed [40,41]. From a practical perspective, there is no way to determine whether data is MAR or not unless follow up data from nonrespondents can be obtained [41]. Without complete follow up data from nonrespondents, it is difficult to determine whether any resulting changes to the outcome analysis results of interest represent a movement toward the “truth” or away from it. Further complicating matters is the fact that missingness alone is not the only potential source of bias in survey results. Obtaining survey data that is inaccurate may be just as problematic [42].

There are several limitations to this study. First, our sample size was small and this study was conducted at a single site. While sufficient to demonstrate proof-of-concept, the small sample size limited study power. Secondly, our findings may not be generalizable to other settings. It is possible that this type of protocol is neither feasible nor cost efficient in larger survey studies where multiple tablet devices (and research assistants) would be required. No additional cost outlay was required in our study as the iPad tablet had already been purchased for research use and a student volunteer functioned as the research assistant. Thirdly, based on our data it is not possible to tease out the impact of the tablet independent from the effects attributable to direct approach as these occurred together. Finally, the fact that non-respondents were more likely to work as part-time or occasional staff is important to note. It is likely that making contact with these individuals in the workplace setting was less probable, limiting the opportunity to invite their participation through direct approach.

### Conclusions

We conclude that use of a strategy involving direct approach with a tablet-based survey is effective in enhancing participant response rate to the desirable 70%-plus level. Further study to assess whether this improved recruitment increases overall representativeness is warranted. Reluctant responders reported positive experiences with this mode of survey administration and no technical issues were encountered.

## Abbreviations

CI: Confidence Interval; OR: Odds Ratio; PED: Pediatric Emergency Department; PCCU: Pediatric Critical Care Unit; PDA: Personal Digital Assistant; Sd: Standard Deviation; Wi-Fi: Wireless Internet.

## Competing interests

Melissa Parker – Dr. Parker is an Assistant Professor of Pediatrics, McMaster University, and a staff physician at McMaster Children’s Hospital. Dr. Parker has received research start-up funding from McMaster and some of these funds may be used if required to cover publication costs in relation to this article. McMaster University and McMaster Children’s Hospital may benefit in reputation from publication of this article. Dr. Parker was granted use of the iSURVEY program for one month free of charge (value $89 USD) to support conduct of this investigator-initiated study.

Asmaa Manan – No competing interests to declare.

Sara Urbanski – No competing interests to declare.

## Authors’ contributions

MP conceived of the idea to utilize a tablet device in an attempt to improve the survey study response rate and to study this prospectively as a nested study. MP developed the protocol, performed the statistical analyses, and wrote the initial draft of the manuscript. AM is a co-investigator in the survey study. She contributed to development of this protocol and the final version of the manuscript. SU assisted with data collection, data entry, and contributed to the final version of the manuscript. All authors read and approved the final manuscript.

## Authors’ information

MP is an Assistant Professor of Pediatrics at McMaster University and an Adjunct Clinical Assistant Professor of Pediatrics at the University of Toronto. AM is currently an MSc student in Biomedical Engineering at McMaster University. SU is currently a BSc student in Life Sciences at McMaster University.

## Funding

MP currently holds research funding in the form of McMaster (MAC) new faculty research start-up grant and as the sponsor for a McMaster Children’s Hospital Resident Research Award. Funds from the McMaster (MAC) new faculty research start-up grant were used to support this work. MP currently receives salary support from McMaster Children’s Hospital and McMaster University. Costs related to use of the iSURVEY program for one month is normally $89.00 USD. The fee for use of this program was waived due to the intent to use this for academic research purposes related to use of mobile technology use in the academic research setting. AM has no funding to declare. SU has no funding to declare. No funding body or institution was involved in the design, collection, analysis or interpretation of the data or in the writing of the manuscript.
